# A classification model for distinguishing copy number variants from cancer-related alterations

**DOI:** 10.1186/1471-2105-11-297

**Published:** 2010-06-02

**Authors:** Irina Ostrovnaya, Gouri Nanjangud, Adam B Olshen

**Affiliations:** 1 Department of Epidemiology and Biostatistics, Memorial Sloan-Kettering Cancer Center, New York, NY, USA; 2 Department of Pathology and Laboratory Medicine, University of California, Los Angeles, CA, USA; 3 Department of Epidemiology and Biostatistics and Helen Diller Family Comprehensive Cancer Center, University of California, San Francisco, CA, USA

## Abstract

**Background:**

Both somatic copy number alterations (CNAs) and germline copy number variants (CNVs) that are prevalent in healthy individuals can appear as recurrent changes in comparative genomic hybridization (CGH) analyses of tumors. In order to identify important cancer genes CNAs and CNVs must be distinguished. Although the Database of Genomic Variants (DGV) contains a list of all known CNVs, there is no standard methodology to use the database effectively.

**Results:**

We develop a prediction model that distinguishes CNVs from CNAs based on the information contained in the DGV and several other variables, including segment's length, height, closeness to a telomere or centromere and occurrence in other patients. The models are fitted on data from glioblastoma and their corresponding normal samples that were collected as part of The Cancer Genome Atlas project and hybridized to Agilent 244 K arrays.

**Conclusions:**

Using the DGV alone CNVs in the test set can be correctly identified with about 85% accuracy if the outliers are removed before segmentation and with 72% accuracy if the outliers are included, and additional variables improve the prediction by about 2-3% and 12%, respectively. Final models applied to data from ovarian tumors have about 90% accuracy with all the variables and 86% accuracy with the DGV alone.

## Background

Copy number variants (CNVs) are a recently discovered part of natural genetic variation in humans. CNVs, also sometimes known as copy number variations or copy number polymorphisms, is a collective term for deletions, insertions, duplications and large-scale copy number variants ranging in size between one kilobase and several megabases [1-4]. About 5-12% of the human genome, including thousands of genes, may be variable in copy number, and this variation can be *de novo *(occurring for the first time in the parent's germ cell) or inherited from the parents by healthy individuals [5,6].

Although their significance is not fully understood, it is likely that CNVs are responsible for a considerable proportion of phenotypic variation. For example, there are established links between CNVs and childhood onset of schizophrenia [7] and autism [8]. It has also been suggested that CNVs can increase the risk of prostate cancer [9] and neuroblastoma [10]. CNVs contribute to the understanding of complex diseases through genome-wide association studies together with SNPs and other types of variants [11,12]. DNA copy number arrays are the main instruments for identifying CNVs. The constant refinement and increasing resolution of these assays is facilitating the discovery and high precision mapping of these variants.

Before the importance of CNVs was realized it was well known that copy number changes occur often in cancer. Copy number alterations (CNAs) are somatic changes in genomic copy number of any size, up to a whole chromosome, that occur in the genome of a cancerous cell. These changes often involve important cancer genes such as tumor suppressor genes and oncogenes. To aid the discovery of important cancer genes, investigators identify regions that are repeatedly gained or lost in patients with a particular cancer. CNVs, present in the genome of every cell, are also present in tumor cells. Thus, when comparative genomic hybridization (CGH) arrays of tumors are studied, both cancer-related CNAs and germline CNVs can appear as unique or recurrent changes. It would be possible to avoid CNVs using a paired tumor-normal design, with the normal tissue from the same individual used as a reference. However, if a paired normal sample is not used or is not available, which is often the case, it is difficult to distinguish CNAs from CNVs. In addition, it is possible that the CNVs in the unpaired reference sample will show up as recurrent events in many or all tumors.

To our knowledge, there are currently no statistical methods to identify CNVs in tumor data. A common practice, recommended, for example, in [13], is to evaluate whether the discovered recurrent regions match known CNVs in the Database of Genomic Variants (http://projects.tcag.ca/variation) [1], which we call the DGV. It is unclear, however, to what extent the regions should match known CNVs. The problems associated with this practice are that it might exclude unnecessarily big regions of the genome, and that regions of exclusion will be imprecise due to uncertainty in the endpoints of the known CNVs [13]. In addition, smaller CNVs are less likely to be included into the DGV since they are detected less frequently in older, low-resolution arrays.

There are other resources available that contain CNVs, for example CNVVdb (http://cnvvdb.genomics.sinica.edu.tw), DECIPHER (https://decipher.sanger.ac.uk/application/), 1000 genomes project (http://www.genome.gov/27528684) and dbVAR (http://www.ncbi.nlm.nih.gov/dbvar/), that eventually may provide a more comprehensive database. However, for simplicity and clarity, we decided to restrict ourselves to only one database and chosen the DGV since it was the most complete and most user-friendly at the time of our analysis. Our goals in this manuscript are twofold. Our first goal is to determine if additional information can be added to that from the DGV for predicting CNVs in cancer data. Our second goal is to determine how to optimally use the DGV for this same purpose. We are looking for any characteristics of CNV regions that are different from cancer-modified segments and thus can be used to improve prediction. We developed statistical models toward this goal. These models, in addition to simplifying data analysis, might allow the discovery of new CNVs from the abundance of available tumor data.

Our models were developed on Agilent 244 K array (AG244) data on glioblastoma (GBM) patients from The Cancer Genome Atlas (TCGA) project [14]. TCGA data are informative because they include many paired tumor and normal tissue samples that have been hybridized in the same manner. Specifically, the tumor samples have been hybridized against a single reference sample, and the matching normal samples have been hybridized against the same reference. Thus, one can identify CNVs in the normal samples as well as distinguish between CNAs and CNVs in the tumor samples. We investigate whether CNVs and CNAs differ in such variables as length, height, loss/gain status, overlap with changes in other patients, and other variables. We divide the patients into training and validation sets, and develop a prediction model that includes these variables and information in the DGV. In addition, TCGA has collected copy number data on several normal tissue samples, which should contain only CNVs, and data from tumor samples co-hybridized with the patient's own normal tissue reference, which should contain only CNAs.We use these data, as well as ovarian TCGA data, to further validate our models.

The outline of the paper is as follows. In the Methods section we describe the data and introduce the types of models that were evaluated. The Results section contains the univariate and multivariate models and their associated prediction accuracies. We conclude with the Discussion.

## Methods

### Selection of patients

We initially selected the first 206 glioblastoma samples qualified for genomic analyses as part of TCGA [14]. All of them were hybridized to the AG244 array as well as other platforms. Here, we limit our analyses to AG244 data collected at the Memorial Sloan-Kettering Cancer Center(MSKCC). There were 78 patients that satisfied requirements for our analysis: paired tumor and normal samples that were independently hybridized against pooled reference. The normal samples varied between blood, skin, and muscle tissue. The patient data were divided into training and test sets by TCGA batch number in order to maintain the heterogeneity that is likely to occur in practice. The training set consisted of TCGA batches 3, 6, and 7, with a total of 43 patients, while the test set contained batch 5 with 35 patients. The raw data are of exceptionally high quality and publicly available at http://cancergenome.nih.gov. The details of the sample selection and preparation were given in [14]. The collection of the original material and data of TCGA study was conducted in compliance with all applicable laws, regulations and policies for the protection of human subjects, and any necessary approvals, authorizations, human subjects assurances, informed consent documents, and IRB approvals were obtained.

We originally chose glioblastoma samples to study CNVs because it was the only set of TCGA patients with a large enough set of matched samples. As it turns out, glioblastoma is a disease where distinguishing germline mutations might prove to be particularly useful because tumor induced alterations are often of similar size. Among heterozygous deletions and amplifications that are present in GBM in at least 10% of patients, as reported previously [15], 40% are focal alterations, and about 90% of the rest of them are under 3 Mb. Altered regions of similar sizes are reported in the TCGA manuscript [14]. In fact its authors excluded regions as CNVs if they 1) appeared to be CNVs in HapMap normals, or 2) appeared in at least 2 independent publications in DGV, or 3) appeared in the matched normal tissue by manual or automated search. These exclusion criteria are complicated and would be difficult to replicate. We developed a model that would simplify such an analysis.

As TCGA progressed some of the data for ovarian cancer became available. We downloaded 38 pairs of matched normal and ovarian tumor samples hybridized at MSKCC. These data were used to validate our models.

### Segmentation analysis

The raw log-ratio data were normalized as described in the supplementary section of the TCGA manuscript [14]. To identify possible regions of gain and loss, we segmented the normalized log ratios using two different algorithms: CBS and GLAD. CBS, or Circular Binary Segmentation [16,17] is a method for segmenting data into regions of equal estimated copy number. It has been found to have good properties compared to other similar methods (see [18,19]), and is included in the Bioconductor package DNAcopy (http://www.bioconductor.org). We have used all default parameters, including the significance level of *α *= 0.01, except for the minimum gap between the segments was set to be one standard deviation (undo.sd = 1). Users frequently apply a smoothing procedure to remove outliers from the data prior to segmentation, which can increase power and remove some smaller gains and losses. On the other hand, smaller regions eliminated by smoothing may contain CNVs. Therefore, we fit the prediction model on both smoothed and non-smoothed data.

Alternatively, we estimated intervals of change using the GLAD algorithm [20], Bioconductor package GLAD) with default parameters. GLAD automatically filters outliers, analogous to smoothing in CBS. The second method was used to ensure that the accuracy of the prediction model was not specific to a particular segmentation method. Both of these methods are frequently used for segmenting cancer data.

### Candidate CNVs

The unit of analysis for us is a segment of constant copy number with breakpoints estimated by one of the segmentation algorithms described previously. We are calling any segment a gain or loss that has an average log ratio of at least one median absolute deviation above or below the array's median, respectively. Segments within this range are called normal.

Suppose only tumor data were available. Then long enough gains and losses would not be confused with CNVs, since CNVs are not longer than several megabases. Likewise, if a segment of interest is in the midst of larger gains or losses, it is difficult to identify whether it was modified in the germline. Therefore, we consider every segment of gain or loss in the tumor that has length of up to 2.3 megabases AND is flanked by at least one normal segment to be a candidate CNV for our classification model. This definition reproduces the situation where the question of identifying CNVs might arise.

Consecutive gains or consecutive losses were combined if their total length was under 2.3 Mb. The upper threshold for length was motivated by the analysis of true CNVs from the normal samples, i.e. 2.3 Mb was roughly the maximum of observed CNVs in the normal samples. Note that CNVs of greater length than this are reported in the literature; however, they comprise less than 1% of reported CNVs, and might have characteristics vastly different from the majority. Although by formal definition CNVs have to be at least 1 kb in length as stated by [3], we did not use this restriction. Since the gap between probes was often large and segment lengths were possibly underestimated, we have included the few segments that were shorter than 1 Kb. Chromosomes X and Y were excluded from consideration. Any candidates located in the "physiological" regions shown in Additional file 1, Table S1 were also excluded following suggestion by [21]. Physiologic CNVs reflect normal somatic rearrangements that occur in the immunoglobulin genes and T-cell receptors during their development [22-24].

**Figure 1:**
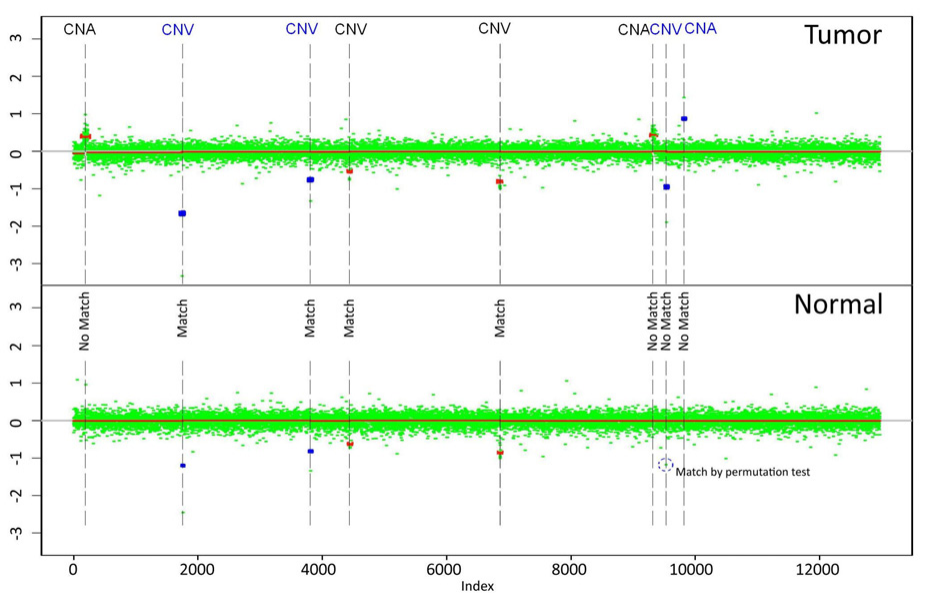
**Example of CNVs and CNAs**. One chromosome is shown. Segments in blue are found only in unsmoothed data. The upper panel contains tumor, while the lower panel is a matching normal sample. CNVs have either a matching segment in the normal sample identified by a segmentation algorithm or matching significantly extreme log-ratios identified by a permutation test. Regions of the normal sample corresponding to CNAs are normal.

The matching normal samples were processed and segmented in exactly the same fashion as the tumor samples. They were used to determine which of the *candidate CNVs *were *true CNVs*. For example, consider Figure 1. All the red segments of gain and loss were found by smoothed CBS on one chromosome; in addition, the blue segments were found if the data were non-smoothed. The top and bottom panels represent a tumor and the corresponding normal tissue. If a gain or loss in a tumor sample exactly matched a gain or loss in a normal sample, it was considered a *true CNV*. However, the segmentation algorithm introduced error in estimation of the breakpoints, so even *true CNVs *might not exactly match between the samples. For example, the red segment marked as CNV overlapped with the matching loss in the normal sample that did not have exactly the same breakpoints.

Furthermore, some segments in the normal sample might not make it to the required threshold of significance and thus would not be identified by the segmentation algorithm. In the figure it can be seen that there is an extremely low log ratio (outlier) in a normal sample corresponding to the last candidate loss (in blue) in the tumor, but it is not extreme or long enough to be identified by CBS. To ensure that such segments were not missed we perform a conditional segmentation test for each *candidate CNV*. In this test we consider log-ratios in the normal sample corresponding to the candidate segment in a tumor. Suppose there are k of them. First we calculate their mean *μ*. Then we randomly draw k values from the pool of normal sample log-ratios that are located within the candidate and two of its neighboring segments in the tumor and calculate their mean this procedure is repeated 1000 times. If the mean of normal log-ratios corresponding to the candidate segment *μ *exceeds the gain/loss threshold and is less extreme than the simulated means μ* in less then (*α *= 0.01 × 100)% of draws, we declare the candidate to be a *true CNV*.

In summary, each gain or loss *candidate CNV *is considered a *true CNV *if it overlaps a gain or loss in a normal sample identified by a segmentation algorithm, or if the mean of the corresponding segment in the normal sample is significant by the conditional segmentation algorithm described above. Otherwise the candidates are considered to be CNAs.

The blue segments in Figure 1 illustrate the trade-offs associated with unsmoothed data. On one hand, one additional CNA and three CNVs emerge when outliers are included in the segmentation. On the other hand, some of these new candidates appear as a result of just one extreme marker. Unless there are array artifacts, it is unlikely that the outliers will simultaneously occur in two arrays in exactly the same probe, and that gives us confidence that unsmoothed data can be useful. However, whenever such data are used the results have to be validated in order to guarantee that the extreme markers are not array artifacts. *True CNV *status for each *candidate CNV *is a response variable in our model. The predictors for our model are discussed below.

### Database of genomic variants

As mentioned before, previously discovered CNVs are reported in the DGV. This database has been used before to distinguish CNAs from CNVs (e.g. [14], but there is no standard quantitative way to make this distinction. In addition, there is some uncertainty in the breakpoints in the reported CNVs. We propose to quantify the overlap with the DGV in the following way. For each probe we calculate the total number of reported variants (unique Variation IDs) that include it. Then, for each *candidate CNV *its *Database score *is defined as average number of these reported variants across all the probes that comprise it. The resulting number is not usually an integer since most overlapping regions in the database do not have the same breakpoints. The less a candidate segment overlaps known CNVs in the DGV, the smaller its *Database score*.

Alternatively, instead of the number of variants we used the total reported number of people that had variants overlapping a probe, and this score is called *Database score II*. This variable is potentially informative because each variant from the DGV has different frequency.

The DGV is updated regularly. For our analysis, we used version 7 of the database for genome build 'hg18' from March 2009 available at http://projects.tcag.ca/variation/downloads/variation.hg18.v7.txt.

### Predictors

Detailed definitions of the candidate predictors for our models are presented in Table 1. These predictors were derived from published results, biological intuition and observations from studying the data. We divide predictors in three categories: "demographic", "derived" and "spatial".

**Table 1:** Definition of predictors.

Variable-Definition
*Length *- length of a segment in bases

*Segmental duplication *- 1 if the candidate is overlapping known region of segmental duplication, 0 otherwise. All regions listed in [32] that could be successfully translated into hg18 by hgLiftOver utility http://genome.ucsc.edu/cgi-bin/hgLiftOver were used, see Additional file 1, Table S2

*Closeness to centromere *- 1 if the candidate endpoints are within 2 Mb of the centromere, 0 otherwise

*Closeness to telomere *- 1 if the candidate endpoints are within 2 Mb of the telomere, 0 otherwise

*Sign *- 1 if the candidate is a gain, -1 if it is a loss

Height - absolute value of the candidate segment mean

*Relative height *- absolute value of the candidate segment mean divided by the median absolute deviation of the array residuals

*Break *- absolute di_erence between means of two segments surrounding the candidate divided by the median absolute deviation of the array residuals

*Surrounded by Normals *- 1 if both surrounding intervals are normals, 0 if one of them is a gain or a loss

*Overlap with other patients *- factor with levels: *GG *if there is one or more other patients in the cohort that have overlapping candidates, all of them are gains; *LL *if there is one or more other patients in the cohort that have overlapping candidates, all of them are losses; *GL *if there are at least two patients with overlapping candidates, some of them are gains and some are losses; *None *if there are no other patients with overlapping candidates

*Overlap with other patients - percent *- proportion of other patients in the cohort that have overlapping candidate

*Matching breakpoint in other patients - percent *- proportion of other patients in the cohort that have a candidate with at least one exactly matching breakpoint

*Close to other candidates *- 1 if there is another candidate CNV within 500 kb on the same chromosome in this patient

*Percent of Normal *- percent of markers on a chromosome where candidate is located that are not lost or gained

*Database score of other candidates *- average *Database score *of other candidates on the same chromosome

*Overlap with CNAs *- number of other patients that have overlapping non-candidate segment of the same sign as the candidate (gain or loss)

"Demographic" variables are the basic characteristics of the candidate segment, such as its length (in bases), absolute value of segment mean (raw or adjusted by the noise level), gain or loss status, and difference between segment means of two nearby segments adjusted by the noise level. We have also included indicator variables for whether a candidate is surrounded by all normal segments, is within 2 MB of centromere or telomere [25], or overlaps any of the known areas of segmental duplication [26]. A "derived" variable uses information from arrays of other independent patients of the same study. The most important of these variables records what percentage of other tumors also have a candidate CNV in the same location, and whether it is a gain or a loss. Since the breakpoints are estimated with error we used two versions of the same predictor, counting other patients with the segments that either overlap the candidate segment or have at least one of the breakpoints exactly matching. If other patients have both gains and losses in the same location, it is likely that this alteration is a CNV. Similarly, a segment that is in an area where large non-candidate gains (or losses) are frequent is likely to be a CNA.

A "spatial" variable captures possible association of CNVs with other segments located on the same chromosome such as percentage of the chromosome that is gained or lost, existence of nearby candidates and average *Database score *of the other candidates on the same chromosome.

### Statistical methods

The response variable in our model is binary, whether a candidate region is a CNV or CNA, while predictors are either binary, multi-level factors, or continuous. To examine the univariate relationship between CNV status and the predictors we utilized univariate logistic regression because it can accommodate variables of all types and provides us with both significance levels and estimates of the effects. Multivariate logistic regression, however, was not used since many of the predictors were highly collinear.

Classification and Regression Trees (CART) is a suitable alternative for highly collinear data [27]. CART is a binary tree approach that is based on recursively splitting on the most predictive variable. CART trees are simple to interpret and have the ability to uncover complex relationships among correlated predictors. One of the disadvantages of CART is that it is often not optimal in terms of prediction error, partly because it is greedy (no looking ahead before splitting). Therefore, we have also used random forests (RF) [28]. RF are a modification of CART that overcome CART's search difficulties by building multiple trees based on resampling cases. Classification is based on the "votes" of each of these trees. These trees further differ from CART trees because only a random set of predictors is considered at each split. Although this algorithm tends to lead to prediction accuracy that is superior to CART [28], the results of RF are more difficult to visualize and interpret. Therefore, we used both CART and RF. CART and RF were implemented using the R packages **rpart **and **randomForest, **respectively. CART models were pruned according to the "1-SE" rule.

All the analyses were performed in R (http://www.r-project.org/) and the final RF models are collected in an RData file, which is available online along with a short manual.

## Results

All the analyses were performed on three data sets: smoothed CBS, GLAD and unsmoothed CBS. In the training data set they contained 1448, 1624 and 2037 candidate segments, respectively, and 904 (62%), 744 (46%) and 1448 (71%) of them were considered true CNVs. The samples in the test set accounted for 1683, 1738 and 2686 candidates and 939 (56%), 846 (49%) and 1727 (64%) true CNVs respectively. There are 638 (510) and 761 (674) segments in the training and test set respectively that are true CNVs (true CNAs) in both smoothed CBS and GLAD, therefore the overlap between smoothed CBS and GLAD is very substantial. The training set contained more patients than the test set but it contained fewer candidate segments. This can be explained by the fact that the training set had slightly noisier arrays (higher MAD of residuals), and, therefore, there was less power to detect smaller segments.

### Univariate results

To test association of predictors with *true CNV *status we pooled the training and test sets. The results within these sets separately were very similar and are not presented. Table 2 contains both Anova p-values and regression *β *coefficients.

**Table 2:** Univariate results by logistic regression, training and test sets combined.

	Smoothed CBS		GLAD		Unsmoothed CBS	
	***β***	**P**	***β***	**P**	***β***	**P**

Height	3.95E - 01	1.16E - 20	4.88E - 01	7.93E - 27	8.88E - 01	6.47E - 117
Relative height	7.14E - 02	3.36E - 17	9.34E - 02	9.75E - 26	1.64E - 01	7.79E - 102
Break	-3.43E - 01	1.06E - 24	-3.39E - 01	2.47E - 26	-3.42E - 01	3.33E - 36
Close to other candidates	-1.19E + 00	2.17E - 32	-8.06E - 01	6.78E - 17	-1.17E + 00	1.19E - 37
Overlap with CNAs	-7.10E - 02	1.24E - 26	-6.67E - 02	6.88E - 25	-5.07E - 02	9.44E - 22
Database score	3.06E - 01	4.08E - 306	3.06E - 01	1.98E - 323	2.27E - 01	8.60E - 186
Database score II	9.79E - 03	3.19E - 159	9.35E - 03	2.16E - 167	5.90E - 03	5.25E - 74
Overlap w. other pts: %	8.89E + 00	4.98E - 254	8.02E + 00	8.64E - 276	5.45E + 00	1.71E - 158
Matching bkpt in other: %	17.31	2.58E - 276	13.13	1.11E - 246	9.14	3.62E - 178
Overlap w. other pts - GG	3.42E - 01	3.63E - 199	3.37E - 01	1.04E - 208	-1.70E - 01	6.08E - 258
LG	2.85E + 00		2.85E + 00		2.29E + 00	
LL	1.68E + 00		1.73E + 00		2.13E + 00	
Closeness to centromere	8.41E - 01	2.55E - 09	7.98E - 01	2.72E - 09	7.15E - 02	5.95E - 01
Closeness to telomere	4.24E - 01	2.15E - 04	5.78E - 01	2.46E - 07	-1.55E - 01	1.27E - 01
Length	-2.46E - 06	2.14E - 130	-1.78E - 06	9.65E - 94	-2.65E - 06	1.27E - 183
Dat. score of other cand.	5.98E - 02	6.69E - 17	5.45E - 02	9.41E - 14	5.17E - 02	9.02E - 11
Percent of Normal	3.40E + 00	5.29E - 59	2.70E + 00	3.35E - 50	2.78E + 00	1.72E - 60
Segmental duplication	6.81E - 01	5.71E - 11	7.16E - 01	1.51E - 13	1.79E - 01	5.85E - 02
Sign	-2.74E - 01	5.10E - 14	-2.62E - 01	2.73E - 13	-7.05E - 01	2.06E - 107
Surrounded by Normals	1.30E + 00	2.28E - 23	1.07E + 00	5.02E - 23	1.31E + 00	7.06E - 32

The smoothed CBS and GLAD had very similar rankings of significant predictors and their effects. As expected, *Database score *had the most significant p-value, followed by *Matching breakpoint in other-patients - percent *in CBS or *Overlap with other patients - percent *in GLAD, *Length*, and *Percent of Normal*. All other predictors were also significant. Obviously, overlap with many variants from the DGV was a strong positive predictor of being a CNV. Segments that were shorter, matched with candidates from many other patients or overlapped with both gain and loss candidates in other patients were also more likely to be CNVs. Having other patients with overlapping candidate losses only was also a positive predictor. As seen from the direction of the main effects in Table 2, CNVs tended to have larger absolute values of segment means; were often surrounded by Normal segments; located on chromosomes with fewer gains and losses, or with other candidates with high *Database score; *or located close to a telomere, centromere or segmental duplication. Also, we saw several clusters of small CNAs right next to each other, so presence of other candidate segments within 500 kb was predictive of CNA.

In unsmoothed CBS the strongest predictor was *Overlap with other patients*, followed by the *Database score*. One possible explanation for this difference is that the small CNVs are underrepresented in the DGV but are likely to appear in the unsmoothed arrays of other patients in the cohort. The other notable difference with smoothed segmentation is that closeness to a centromere, telomere or segmental duplication were not significant, possibly because longer CNVs tend to be located there. In fact, the interaction term between length and closeness to centromere (or segmental duplication) was significant in logistic regression for both smoothed and unsmoothed CBS. As demonstrated by the interaction effect, segments at these locations and of longer length were even more likely to be CNVs.

Note that these associations are not causal, and the mechanisms by which CNVs occur and fixate in the population are still to be elucidated.

### Prediction models

**Figure 2:**
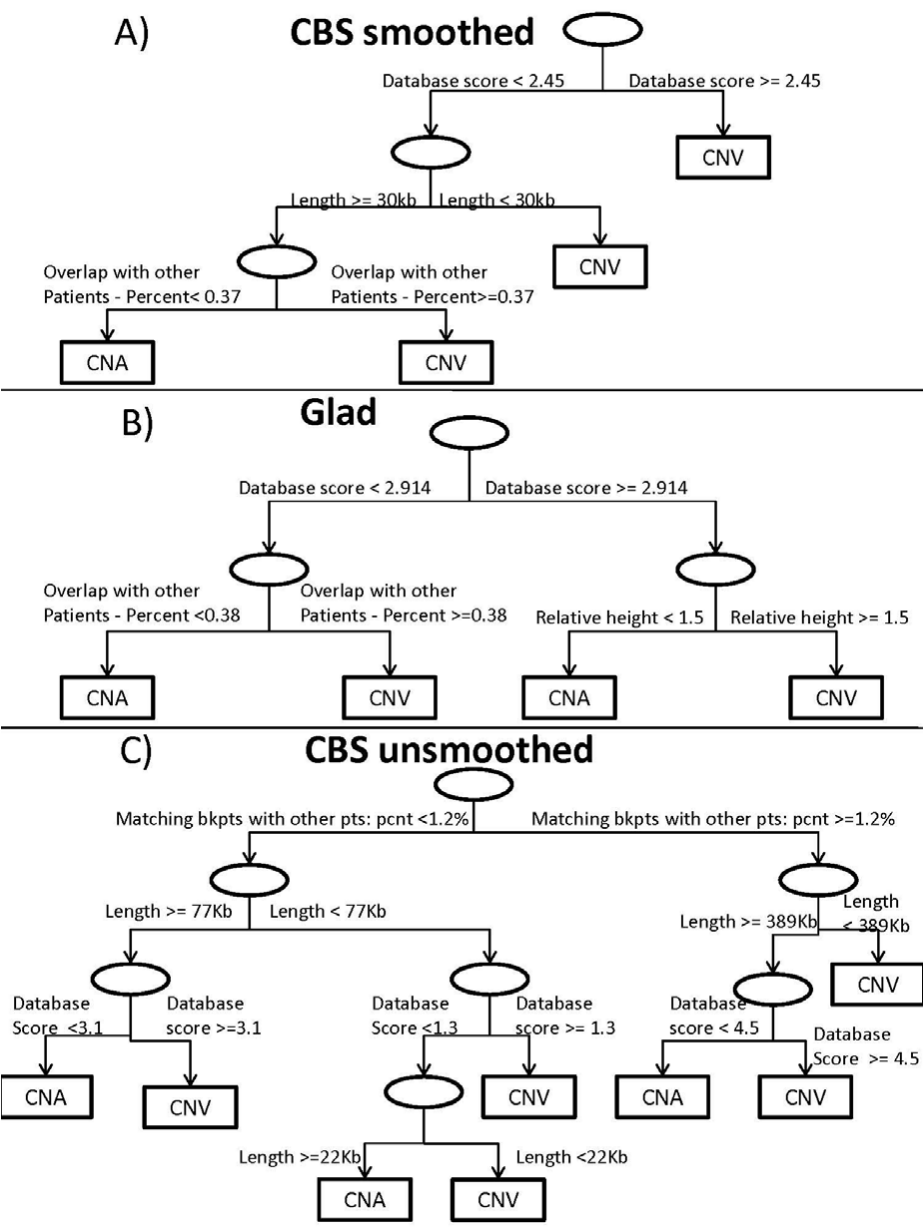
**Fitted CART models**.

We have fitted prediction models using smoothed CBS, GLAD and unsmoothed CBS with 4 different sets of predictors. Accuracy was defined as the percentage of correctly classified candidate segments among all CNV candidates in all tumor samples of validation set. Accuracy was evaluated on 3 validation sets. The full set of predictors contained all the variables described in Table 2 except *Database score II *and *Height *that were nearly equivalent to the already included variables *Database score *and *Relative height*. We first will discuss the results based on smoothed CBS. The fitted CART model selected only five predictors, as is shown in Figure 2. The first split was made on the *Database score: *if the probes in the candidate segment were included in the DGV at least 2.45 times on average, the candidate was predicted to be a CNV. Otherwise, only segments with the following characteristics were predicted to be CNVs: 1) segments shorter than 30 Kb; or 2) segments of length longer than 30 Kb that had matching candidate segments in 37% or more of the other patients. The prediction accuracy of this model, estimated for the smoothed CBS test set, was 86%, as shown in Table 3. Table 4 has the numbers of candidate segments that were correctly and falsely classified. There were 793 true CNVs predicted to be CNVs, and 654 correctly identified true CNAs. Interestingly, number of CNAs falsely identified as CNVs (182) was much higher than number of missed CNVs (54). We believe CNVs were easier to identify because the DGV contains extensive information about them.

**Table 3:** Prediction rates: A - test set, B - CGH against self-reference (all CNAs), C - normal tissue (all CNVs).

	CBS smoothed			GLAD			CBS unsmoothed		
	A	B	C	A	B	C	A	B	C
CART-full model	0.86	0.79	0.90	0.83	0.91	0.78	0.80	0.66	0.92
RF - full model	0.87	0.82	0.95	0.86	0.91	0.87	0.84	0.77	0.92
CART- database only	0.85	0.88	0.81	0.83	0.89	0.80	0.72	0.34	0.99
RF - no database	0.85	0.79	0.94	0.86	0.89	0.85	0.82	0.74	0.95
RF - one array	0.85	0.81	0.97	0.84	0.91	0.89	0.83	0.75	0.96

**Table 4:** Counts from the accuracy table of the test set.

	CBS smoothed				GLAD				CBS unsmoothed			
	TN	FN	FP	TP	TN	FN	FP	TP	TN	FN	FP	TP
CART-full model	654	54	182	793	822	145	157	614	613	77	455	1541
RF - full model	659	42	177	805	828	93	151	666	752	124	316	1494
CART- database only	699	120	137	727	804	122	175	637	364	37	704	1581
RF - no database	644	57	192	790	824	91	155	668	686	112	382	1506
RF - one array	647	59	189	788	832	125	147	634	729	120	339	1498

(TN, True Negatives, are true CNAs predicted to be CNAs; FN, False Negatives, are true CNVs predicted to be CNAs; FP, False Positives, are true CNAs predicted to be CNVs; TP, True Positives, are true CNVs predicted to be CNVs)

The two other validation sets we used were the set of 39 tumors that were hybridized against self-reference, so that all its 1780 candidates were considered true CNAs, and the set of 8 normal tissue arrays, so that all its 257 candidates were considered true CNVs. As seen in Table 3, 79% and 90% of these segments, respectively, were identified correctly. As in the test set, the rate of missed CNVs was smaller.

The RF model with the same set of predictors increased the accuracy by 1% on the test set, and by 3-5% on the 'all CNAs' and 'all CNVs' sets. Since the best model was a combination of many trees it is difficult to display; however, relative importance of each variable measured by Gini index is shown in Table 5. The more influential variables have higher indices. The ranking of the variables was roughly consistent with univariate results: the top predictors were *Database score, Length, Matching breakpoint in other patients -percent, Overlap with other patients - percent, Percent of Normal *and *Relative height*.

**Table 5:** Relative importance of variables in random forest models as measured by Gini index (higher is more important).

	CBS smoothed			GLAD	CBS unsmoothed	
Variable	w. DS	w/o DS	w. DS	w/o DS	w. DS	w/o DS
Relative height	60.33	79.86	81.26	100.96	101.99	119.94
Break	39.98	51.92	48.57	65.75	60.33	72.19
Close to other candidates	8.27	10.83	4.18	6.90	6.19	8.20
Overlap with CNAs	17.21	24.15	17.87	27.10	26.16	34.09
Database score	165.44		206.34		108.89	
Overlap w. other pts: %	70.11	107.05	95.23	129.03	74.62	93.67
Matching bkpts in other: %	86.67	108.45	94.02	135.80	116.34	116.83
Overlap with other pts	39.91	59.70	42.18	68.84	86.42	98.28
Closeness to centromere	4.37	5.82	3.74	6.07	4.64	7.17
Closeness to telomere	4.65	6.53	3.31	5.52	6.04	7.11
Length	112.20	133.69	64.26	92.25	170.78	182.46
Dat. score of other cand.	30.23		32.57		44.51	
Percent of Normal	64.10	84.05	55.97	76.26	77.18	93.82
Segmental duplication	3.19	7.77	3.48	9.53	5.16	9.34
Sign	7.84	11.01	8.19	12.42	22.59	27.77
Surrounded by Normals	1.87	4.67	5.03	6.66	3.34	5.21

"'W. DS"' stands for the model that includes *Database score *(DS), "'w/o DS"' stands for the model where it was excluded.

We have also fitted the CART model with a single variable - *Database score*. Its only split was the same as the first split of the full model: segments seen in the DGV on average 2.45 times were predicted to be CNVs. The prediction accuracy of this model was equal to 85%, 88% and 81% on the test, 'all CNAs' and 'all CNVs' sets respectively. Therefore, using all the proposed predictors on the test set in addition to the *Database score *increased the accuracy on the test set by 2%. As can be seen from Table 4, the sensitivity of the full RF model is 82%, which is slightly lower than the 84% sensitivity of the *Database score *only model. The specificity, however, of the full RF model is much higher: 94% compared to 85%.

The fitted CART tree was different using GLAD, although the first split was still made on the *Database score*. As seen in panel (b) of Figure 2, segments were predicted to be CNVs if 1) they were included in the DGV at least 3 times on average and had relative absolute mean greater than 1.5; or 2) they were included in the DGV less than 3 times on average and overlapped with other candidates in at least 38% of other patients of the cohort. In the RF model for GLAD, the 6 variables with the highest Gini indices (Table 5) were the same as in smoothed CBS, while having slightly different ranking. In spite of these differences, the prediction characteristics of models based on GLAD and smoothed CBS were similar. The RF with all predictors correctly identified 86% of candidates in the test set, 91% of 1861 true CNAs in 'all CNA' dataset, and 87% of 247 true CNVs in 'all CNVs' set, while the rates of false CNVs and false CNAs were more balanced. The model with only *Database score *had the same first split of *Database score *greater or less than 3, and its accuracy on the test set was only 3% smaller than that for the full model. For GLAD, the sensitivity was 82% and 78% for the full RF and *Database score *only models respectively, while the specificity was 90% and 87%.

Since many of the predictors were highly correlated there could be many classification trees with similar prediction accuracy, so the difference in models between GLAD and smoothed CBS might be a result of random variation rather than fundamental segmentation differences. In fact, when we applied the full RF developed on the GLAD segmented training set to the smoothed CBS test set, the prediction accuracy was 87%, which was the same as the model developed based on smoothed CBS. Similarly, the RF developed on the smoothed CBS training set resulted in 83% accuracy when assessed on the GLAD test set, which was just 3% lower than the RF developed on the GLAD training set.

Prediction modeling based on unsmoothed CBS had several important differences. The variable *Matching breakpoint in other patients - percent *served as the first split in the classification tree. If a segment 1) had candidates with matching breakpoints in more than 1.2% of other patients and was either shorter than 396 Kb or was both longer than 396 Kb and was included in the DGV on average 4.5 times; or 2) had candidates with matching breakpoints in less than 1.2% of other patients, was shorter than 22 Kb, or shorter than 77 Kb and included in the DGV on average 1.3 times, or longer than 77 Kb and included in the DGV on average 3.1 times, then it was predicted to be a CNV. Note that in our training set the first split is equivalent to having at least one other tumor with breakpoint exactly matching the breakpoint of a candidate. RF had the same 6 variables with the highest Gini indices as two other segmentation methods, and it correctly predicted 84% of segments in the test set, as well as 77% of 1785 CNAs and 92% of 464 CNVs in the two other validation sets. Unlike in smoothed CBS and GLAD, the classification tree that included only *Database score *showed only 72% accuracy on the test set, 12% lower than the full model. The sensitivity was 83% and 69% for the full RF and *Database score *only models respectively, while the specificity was 86% and 91%. This model had a much higher false CNV rate - 66% of all CNAs in the test set and 66% of the CNAs in 'all CNAs' validation set were falsely identified as CNVs (see Tables 3, 4). We speculate that unsmoothed CBS contained smaller intervals that rarely appeared in the DGV, and the *Database score *was less informative about them. As a result the model had lower prediction rates on the validation sets.

While the DGV provided the strongest univariate information, we investigated whether it was absolutely necessary for predicting CNVs by fitting RF that excluded *Database score *and *Database score of other candidates*. We saw only a modest drop in prediction accuracy of 0-2%. The most important variables suggested by the Gini index *Matching breakpoint in other patients - percent, Overlap with other patients -percent, Length, Relative height*, and *Percent of Normal *were the same across all three segmentation methods.

Since *Overlap with other patients *and *Overlap with CNAs *are only informative when there are multiple patients in the cohort, we have also considered models without these variables since they could be applied to single arrays. They are presented in the last row of Tables 3 and 4. There was a 1-2% loss of accuracy compared to the full models.

The accuracy measure in Table 3 represents per-study error rate where all potential CNVs in all tumors are pooled together. Note that CNVs that appear in many patients will have a higher chance of being correctly classified compared to the rare CNVs. Thus, per-variant accuracy might be lower on average than per-study accuracy. We do not estimate the per-variant error rate since it is not clear how to classify observed segments into distinct variants. Another accuracy metric we considered was per-tumor accuracy. The median per-tumor accuracy rates are shown in the Additional file 1, Table S3 and are very similar to per-study accuracy rates.

Since, as we mentioned, training set had slightly noisier arrays, we have also examined whether switching training and validation sets would lead to different results. The accuracy rates were in fact similar to those shown in Table 3. For example, the accuracy of the full RF model and model with *Database score *only developed on the former testing set and tested on the former training set, was 86% and 81% respectively for smoothed CBS, 88% and 84% for GLAD, and 88% and 78% for unsmoothed CBS. Thus, the full model gives similar advantage compared to the *Database score *only as seen previously.

While the basic CART model with just *Database score *works fairly well, we have tested it against the basic ad-hoc rule often used in the literature: a candidate CNV is classified as CNV if it overlaps variants reported in the DGV from at least two different studies (manuscripts), and it is a CNA otherwise. Based on the combined training and testing set (there is no model development), this ad-hoc rule identified correctly 65%, 62% and 60% candidates in smoothed CBS, GLAD and unsmoothed CBS respectively, significantly fewer than CART *Database score *only.

### Validation using ovarian dataset

We expect the models to be valid across all cancers as long as few CNVs are associated with cancer. To verify this we obtained 38 pairs of matched normal and ovarian cancer samples from the TCGA website. They were segmented using smoothed CBS only, the method we predominantly use in practice. Since they were already smoothed during the normalization process, unsmoothed segmentation was not performed. There were 2623 candidate CNVs in the tumor samples of which 485 were called true CNVs by the same algorithm that was used for glioblastoma data. The models developed based on smoothed CBS and GLAD segmentation have prediction accuracy of 86% and 87%, respectively, if they contain the DGV information only, and 89% and 90% if all predictors are utilized. As in the GBM data, the error rates of the full smoothed CBS model are unbalanced: 230 CNAs predicted as CNVs and 70 missed true CNVs, with 1908 and 415 correctly identified CNAs and CNVs. The errors are well balanced for the GLAD-derived model; the respective counts are 134, 137, 2004 and 348. Thus, a model derived from GLAD-segmented glioblastoma data offers about 90% prediction accuracy, which is 3% higher than the DGV only model, as well as balanced error rates, even in ovarian cancer data.

## Discussion

In this article we introduced a framework for distinguishing germline copy number variants (CNVs) from cancer-related copy number alterations(CNAs) when analyzing tumor samples on copy number arrays. To our knowledge, our manuscript is the first attempt to quantify the overlap of a given copy number abnormality with the database of genomic variants (DGV) and to suggest a rule for determining CNVs. We have also examined various characteristics of the altered segments that can differ between CNVs and CNAs. We considered three segmentation methods to identify candidate CNVs and built CART and RF prediction models using up to 16 predictors that can be applied to both cohorts of several independent patients and to single arrays. If the segmentation was done after removing outliers then the most important predictor was overlap with DGV. If each probe of a candidate segment overlapped on average with 2.5 - 3 variants listed in the DGV, this candidate segment was likely to be a CNV. Inclusion of additional variables like *Length, Relative height, Overlap with other patients in the cohort *improved the accuracy by a few percent. The model developed using one segmentation method can be successfully applied to another equivalent segmentation method (smoothed CBS and GLAD). The advantage of additional predictors was more pronounced (12% higher accuracy) if the segmentation was performed on data with no outliers removed. Such data were more likely to contain smaller candidate segments that are missed in the DGV.

Overall, the prediction accuracy in the test set is around 85% across different segmentation methods. We have also applied the classification algorithm to validation sets containing only CNVs (normal samples) or only CNAs (tumor samples with CNVs subtracted as a reference). The candidate segments were correctly classified in these datasets in 80-95% of cases, even though our classification model was not developed on these types of samples. The prediction accuracy of the full model evaluated on the smoothed CBS ovarian data set was 90%.

Note that the variable *Database score*, while being one of the most significant, is based on the DGV, which has some inaccuracies and repetitions. For each probe it involves the count of variants listed in the DGV that covered that probe. These variants, however, are not independent and the representation of many of them in the DGV is redundant. For example, many studies report identical or almost identical variants observed on the same sets of patients (e.g. HapMap patients), and a few variants are listed twice even within the same study. In addition, variants have been observed in a different number of people and have different frequencies, although our variable *Database score II *that utilized these frequencies did not prove to be superior, probably also due to redundancy in variant reporting. All other modifications of the Database scores that we tried, including a score that used the percentage of people that exhibited the variant in each study, did not offer the improvement in the prediction rates. Note that CNVs discovered by fine scale mapping of DNA from HapMap patients [29] were much smaller, often by more than 50%, than CNVs reported in the DGV based on previous studies. Also, many variants that were discovered using lower resolution assays like BAC arrays were likely to have inaccurate endpoints; however, excluding BAC arrays from the calculation of the *Database score *did not improve the prediction. As the DGV and the frequencies of all known CNVs become more accurate, it may be possible to improve the prediction model. Although we have identified *true CNVs *by matching the candidate segments in tumors to their corresponding normal samples, our classification is not a gold standard. Due to stromal contamination, segmentation error and possibly imperfect gain/loss calling, some CNVs and CNAs in the tumors might have been missed. It is also plausible that some true CNVs were missed by our matching method and classified as true CNAs. However, since we have verified the prediction framework on several validation sets, we do not expect the error in true CNV classification to have had a major impact on the model. We also believe that the fitted models are not specific to glioblastoma since CNVs should be mostly homogeneous across patients with different cancers, and the good predictive ability on the set of ovarian cancer samples and on the normal samples supports this claim.

A possible feature that could help identify CNVs is ethnicity. We know that CNV proportions vary by ethnic group [30]. However, the early TCGA data on which we have built our models is mostly Caucasian, so it is not currently possible to use ethnicity. One mitigating factor is that the DGV includes CNVs from many populations, and variables (e.g. length, height) that differ between cancer CNAs and CNVs in Caucasians would be expected to have the same relationships across ethnic populations.

Our models do not depend on the scale of log-ratios since the only important predictor that depends on them, absolute segment mean, is divided by the median absolute deviation of noise and as a result, *Relative height *is scale and noise level invariant. Nevertheless, all our analyses are done on Agilent 244 K arrays, and CNVs might have different characteristics if they were detected on arrays of different resolution or different platforms. We believe that the model will be as efficient on arrays that have similar or worse quality and resolution, since they likely identify CNVs that are similar to or a subset of what can be found by the Agilent 244 K platform.

The Agilent 244 K array, like all non-SNP arrays, measures total copy number rather than allele-specific copy number. That is, it cannot separately estimate the two parental copy number contributions. This could lead to occasional error in our analysis. For instance, if there were CNVs on both alleles, and if there were a somatic copy neutral LOH event (one parental copy number doubles while the other disappears) that was larger than the CNV event, it is possible that we would interpret this event as a CNA (which would be correct), a CNV, or a normal region depending on the combination of copy numbers on two alleles in the CNV. The interpretation would depend on the allelic copy numbers of both the normal and tumor samples. This problem, however, is due to the limitation of the array, not to our algorithm.

Many studies that have the goal of identifying cancer genes deliberately exclude CNVs prior to analysis (e.g. [14]. One way to do this using our algorithm would be to segment the original data and apply the appropriate RF model. The probes within predicted CNVs in at least one, or, conservatively, several patients could be excluded, and the reduced data set could be segmented again for the final analysis. Alternatively, since predictions are obtained for all segments that are located in areas of suspected recurrent gain or loss, a region might be discarded if some or many of the matching candidates are predicted to be CNVs.

It is also interesting to study CNVs in cancer patients. For example, there is evidence that CNVs may contribute to chromosome breakage [31] and to cancer risk [9,10]. There are abundant studies of copy number on cancer patients that are publically available. For any of these studies the data can be segmented and all the candidate segments can be classified as CNVs or CNAs. Assuming no CNVs from the reference sample appear in the tumors, the presence of CNVs as identified by the proposed method can be correlated with recurrent CNAs or clinical characteristics. Therefore, the classification model that we developed may facilitate the study of the associations between CNVs and cancer predisposition or progression.

## Conclusions

We have developed several prediction models that distinguish germline copy number variants (CNVs) from cancer-related copy number alterations (CNAs) based on copy number arrays of tumor samples with no matching normal sample. Using the Database of Genomic Variants alone CNVs in the test set can be correctly identified with about 85% accuracy if the outliers are removed before segmentation and with 72% accuracy if the outliers are included, and additional variables improve the prediction by about 2-3% and 12%, respectively. Final models applied to data from ovarian tumors have about 90% accuracy with all the variables and 86% accuracy with the DGV alone. Based on the accuracy rates, we recommend the full RF model developed using unsmoothed CBS to identify CNVs for datasets that contain outliers, while we recommend the full RF model developed using GLAD for datasets in which the outliers have been removed.

## Authors' contributions

IO and AO designed the method and drafted the manuscript. GN contributed to the interpretation and discussion. All authors have read and approved the final manuscript.

## Supplementary Material

Additional file 1 **Supplementary tables**. Additional file 1 consists of three supplementary tables. Table S1 contains list of physiological regions that were excluded from analysis. Table S2 contains regions of segmental duplication (hg18). Table S3 contains median prediction rates within each tumor: A - test set, B - CGH against self-reference (all CNAs), C - normal tissue (all CNVs). Click here for file
