# Supplementary material for: A classification model for distinguishing copy number variants from cancer-related alterations
Source: BMC Bioinformatics. 2010 Jun 2;11:297. doi: 10.1186/1471-2105-11-297 (PMC2897829; doi:10.1186/1471-2105-11-297)
Supplement: Additional file 1 — Supplementary tables. Additional file 1 consists of three supplementary tables. Table S1 contains list of physiological regions that were excluded from analysis. Table S2 contains regions of segmental duplication (hg18). Table S3 contains median prediction rates within each tumor: A - test set, B - CGH against self-reference (all CNAs), C - normal tissue (all CNVs). [file 1471-2105-11-297-S1.PDF]

Additional Files

Table S1: excluded physiological regions

Chromosome	Band	Start	End	Length (Mb)
14	14q32	88900000	106368585	17.47
2	2p11	83700000	93300000	9.60
22	22q11	11800000	24300000	12.5
14	14q11	15600000	23600000	8.0
7	7p15-14	19500000	43300000	23.8
7	7q35	142800000	147500000	4.7

Table S2: regions of segmental duplication (hg18)

Chr	Start	End	Chr	Start	End
1	16570674	21529374	11	58554872	58637894
1	39644292	39911443	11	67234307	71193473
1	47099424	47334837	11	89115225	89470331
1	103844412	104029488	12	9327471	9492133
1	146602113	146661002	12	62209686	62432514
1	193443399	193652009	13	18671435	24453912
1	219038488	224480378	13	51669868	52085380
3	125158410	127197788	13	63188926	63316389
3	196868577	198872158	14	23493142	23575846
4	69859408	70069921	14	105115790	105315245
4	119878950	120719095	15	18458590	30687000
4	145064435	145427952	15	32458295	32663190
5	288807	1670110	15	41638428	41829002
5	98754230	99764829	15	42896927	43162682
6	26775196	26910469	15	70698861	76014417
6	167549682	167776550	15	80369380	83616515
7	5706510	6645758	15	98136837	100152120
7	29465820	34905805	16	11926088	30254369
7	39589900	56270708	16	68535000	73147662
7	63915737	64811465	17	2900905	3103469
7	65881831	76336440	17	25951597	27440083
7	101575559	101918082	17	40928985	42496803
7	142735699	143009437	17	55005024	55434745
7	143321690	143512024	17	55438128	57730605
7	149024431	153302009	18	10594202	12221380
8	2167585	2331389	19	12357800	12416157
8	6933975	12586975	19	22351818	22654242
8	145259369	145464193	19	41455485	42488459
9	33513535	38611353	19	48141176	48323040
9	83653430	85697014	19	48390180	48493611
9	87757240	87984665	19	48532243	48593758
9	90061677	90604904	19	53098556	55818494
9	94148624	96869327	20	45887213	45968382
10	15014850	15108910	21	14268806	14363850
10	42509728	44964465	22	15380222	23404567
10	80921582	82008421	22	23947411	24252773
10	88972008	89250343	22	41226611	41302954
10	135125032	135282940			

Table S3: median prediction rates within each tumor: A - test set, B - CGH against self-reference (all CNAs), C - normal tissue (all CNVs)

	CBS smoothed			GLAD			CBS unsmoothed		
	A	B	C	A	B	C	A	B	C
CART-full model	0.87	0.76	0.91	0.82	0.91	0.81	0.82	0.67	0.94
RandomForest - full model	0.87	0.81	0.95	0.86	0.90	0.95	0.85	0.75	0.92
CART- literature only	0.85	0.88	0.81	0.83	0.89	0.83	0.75	0.34	1.00
RandomForest - no literature	0.84	0.75	0.97	0.86	0.86	0.86	0.84	0.71	0.94
RandomForest - one array	0.86	0.80	0.98	0.84	0.91	0.95	0.84	0.74	0.95
